# Management of Pediatric Foreign Body Injuries during the COVID-19 Pandemic: Results of an International Survey

**DOI:** 10.3390/children10121845

**Published:** 2023-11-24

**Authors:** Honoria Ocagli, Danila Azzolina, Andrea Francavilla, Emrah Aydin, Solidea Baldas, Alejandro Cocciaglia, Hugo Rodriguez, Dario Gregori, Giulia Lorenzoni, Maayan Gruber, the COVID-19-Susy Group

**Affiliations:** 1 Unit of Biostatistics, Epidemiology and Public Health, Department of Cardiac, Thoracic, Vascular Sciences, and Public Health, University of Padova, 35122 Padova, Italy; honoria.ocagli@unipd.it (H.O.); danila.azzolina@unife.it (D.A.); andrea.francavilla@studenti.unipd.it (A.F.); giulia.lorenzoni@unipd.it (G.L.); 2 Department of Medical Science, University of Ferrara, 44121 Ferrara, Italy; 3 Department of Pediatric Surgery, School of Medicine, Koç University, 34450 Istanbul, Turkey; emrah.aydin@nku.edu.tr; 4 Protecting Children Association (Prochild) Onlus, 34129 Trieste, Italy; solideabaldas@prochild.eu; 5 Sanatorio Mater Dei, Buenos Aires C1425, Argentina; endoscopiaresp@garrahan.gov.ar; 6 Hospital de Pediatría Juan P. Garrahan, Buenos Aires C1245, Argentina; harodriguez@garrahan.gov.ar; 7 Galilee Medical Center, Azrieli faculty of Medicine, Bar-Ilan University, Haifa 22100, Israel; maayang@gmc.gov.il

**Keywords:** COVID-19, foreign body, Susy Safe network, CAS

## Abstract

The COVID-19 pandemic has had direct and indirect effects on daily life. In hospitals, the impact of the pandemic was observed in the diagnostic and therapeutic workflow. In this work, we explored potential changes in activities related to the treatment of foreign body injuries (FBIs) in children and the behavioral habits of physicians during the first wave of the pandemic. An online survey was conducted among physicians of the Susy Safe network. The survey comprised items related to respondent information, reference center characteristics, the treatment of FBIs during the COVID-19 pandemic, and a modified COVID-19 Anxiety Scale (CAS). The survey was distributed among the Susy Safe project international network surveillance registry for FBIs. A total of 58 physicians responded to the survey, including 18 (32%) from Europe and 16 (28%) from South America. The respondents indicated that the estimated number of aspirated foreign bodies during the pandemic was lower than or the same as that before the pandemic (43, 74%), and the same was observed for ingested foreign bodies (43, 74%). In univariable logistic regression, no single predictor was associated with a delay in routine care for children or an increasing tendency of medical personnel to avoid procedures. The workflow of physicians involved in the management of FBIs in children has not changed drastically during the COVID-19 pandemic, especially in emergency departments.

## 1. Introduction

The COVID-19 pandemic has directly and indirectly affected people’s daily lives [1]. Although effective in limiting virus spread, containment strategies have affected everyday life, with profound modifications to individuals’ usual lifestyles, including their eating habits [2]. Furthermore, prolonged stress due to public health policies aimed at limiting the spread of COVID-19, such as total and partial lockdowns, isolation, and social distancing, has been well documented to substantially impact mental health [3,4].

In the early stages of the COVID-19 pandemic, it was evident that health care workers (HCWs) faced a considerable risk of contracting COVID-19 [5]. Among medical doctors (MDs), otorhinolaryngologists were particularly affected, as acknowledged by the Confederation of European Otorhinolaryngology-Head and Neck Surgery [6]. This higher risk may have been attributed to their close contact with the airway mucosa of patients during routine ear–nose–throat (ENT) examinations, such as endoscopy and upper airway surgeries, which may induce sneezing and coughing in patients. Given the high viral load of SARS-CoV-2, especially in the nasopharynx, COVID-19 presents an increased risk for otorhinolaryngologists [7].

Along with the risk of contracting COVID-19, many effects on mental health have been reported in the general population since the start of the pandemic. Fear and anxiety about COVID-19 have been proposed to be related to a new specific condition called “coronaphobia” [8], which has been classified as a predictor of psychological distress [9]. Furthermore, the psychological impact of the pandemic has relevant consequences for health care workers [10], especially those who care for patients with COVID-19. These professionals are at increased risk of experiencing anxiety, depression, and insomnia [11]. In a cross-sectional study by Alshekaili et al. [12], front-line health care workers were approximately 1.5 times more likely to report anxiety than non-front-line health care workers. Otorhinolaryngologists were among these front-line health care workers.

A systematic review and meta-analysis by Mogharab et al. [13] revealed a significant disruption in the referral of patients for emergency medical care during the COVID-19 pandemic. This disruption emphasizes the urgent need to address delayed patient-related care as a major health concern. Similar challenges were observed in cases of myocardial infarction (AMI) and heart failure (HF) during the pandemic [14]. Delayed access to health care and a reduction in the number of visits to the emergency department [15] and hospitalizations [16,17], except for domestic accidents, involving any type of disease or injury, including foreign body (FB) injuries, have been reported [18,19]. A case report showed a late diagnosis for a patient after battery ingestion [20] and another for a patient with a fishbone in their larynx [21]. With the reduced mobility of subjects due to lockdown, fear of contagion was claimed to be responsible for the documented reduction and delays in emergency department visits for children and adults. This trend extended to acute appendicitis in children during the pandemic, where a higher incidence of complicated cases and increased utilization of nonoperative management were observed [22]. However, in the case of testicular torsion in children, Pogorelic’s study found no significant differences in symptom duration, delayed presentation, or orchiectomy rates between the pre-COVID-19 and COVID-19 periods [23].

The present work aimed to explore the changes in the management of FB injuries in the first wave of the pandemic and to assess the effects of the COVID-19 pandemic on the activities of otolaryngologists (ENTs) involved in FB injury management in pediatric patients. The current survey centered around the perceived changes and effects resulting from the COVID-19 pandemic among physicians involved in the management of pediatric foreign body injuries.

## 2. Materials and Methods

The primary endpoint in the study was the perceived change in the management of foreign body injuries during the COVID-19 pandemic. The secondary endpoint was the perceived impact of the COVID-19 pandemic on otolaryngologist (ENT) anxiety in the context of foreign body injury management in pediatric patients.

### 2.1. Study Design and Participants

An online survey was conducted between 9 March and 3 May 2020, during the first wave of the COVID-19 pandemic. The survey was conducted through the REDCap web application [24] and distributed to Susy Safe network physicians. The Susy Safe project is an international surveillance registry for injuries due to the ingestion, aspiration, inhalation, or insertion of organic and inorganic foreign bodies in children [25]. Cases collected in the registry are reported anonymously by health care professionals, including physicians, ENT experts, pneumologists, pediatricians, and general practitioners who are part of the project’s network. Invitations to participate in the survey were distributed via email to all investigators collaborating on the Susy Safe project. Additionally, reminders to complete the survey were periodically sent out throughout the project’s duration. A total of 172 physicians received the invitation to participate in the survey.

### 2.2. Procedure

Participants completed a questionnaire online. They were asked to provide their informed consent before participating in the survey. The questionnaire consisted of four parts collecting general information, that is, the number of years of experience, characteristics of the center, nation, average number of patients managed in one week, number of medical and health care professionals working in the center, and management of FB injuries during the COVID-19 pandemic period. Finally, the COVID Anxiety Scale (CAS) questionnaire was administered [9].

### 2.3. COVID-19-Related Questions Structure

The following domains were examined: (i) the treatment of FB injuries (whether the estimated number of foreign bodies ingested or aspirated was the same (higher, lower, same); differences in the type of FB (yes/no); delay of foreign body presentation (yes/no); delay in the workup of the hospital (yes/no); different workup methods (yes/no); the use of CT scans (yes/no); the influence of COVID-19 test results; and the avoidance of procedures (yes/no)); (ii) the procedures for FB removal (availability of personal protective equipment (PPE) during procedures (yes/no); use of PPE during procedures (yes/no); procedural complications due to PPE (yes/no); delayed timing of procedures (yes/no); change in protocols for anesthetizing patients (yes/no); differences in surgical techniques (yes/no); presence of a higher number of complications (yes/no)); (iii) the procedures after FB removal (checking COVID-19 status after procedures (yes/no); awareness of performing such procedures on COVID-19-positive patients (yes/no); COVID-19 positivity among the respondents and staff (yes/no)); and (iv) CAS scores.

### 2.4. The CAS Questionnaire

The CAS questionnaire is a validated instrument developed by Lee to ‘identify probable cases of dysfunctional anxiety associated with the COVID-19 crisis’ [9]. The CAS questionnaire is a 5-item instrument that is highly reliable (Cronbach alpha: 0.93) with good diagnostic properties (90% sensitivity and 85% specificity) [9]. A score ≥ 9 indicates dysfunctional anxiety [26,27]. Participants rate how often they experience anxiety-related symptoms using a 5-point Likert-type scale (0 = not at all to 4 = almost every day in the last two weeks) [27]. We used a modified version of the instrument in this study, with the same questions related to cases of FB injury during the COVID-19 pandemic.

### 2.5. Statistical Methods

Descriptive statistics are reported as medians (I and III quartiles) for continuous variables and percentages (absolute numbers) for categorical variables. The Wilcoxon/Kruskal–Wallis test for continuous variables and the Pearson chi-square test for categorical variables were performed for group comparisons. Normality tests were not conducted because it is well established that the results of normality tests can be influenced by sample size. Larger sample sizes may yield statistically significant results for even minor deviations from normality, whereas smaller samples might not detect such deviations [28,29,30].

In situations such as this, nonparametric tests prove to be robust when the assumption of normality is violated. They do not rely on specific underlying distributions, making them versatile for various data types, including those that may not adhere to a normal distribution. Particularly in cases of small sample sizes where the data may not follow a normal distribution, nonparametric tests can provide reliable results without the necessity for normality testing.

The questions “Did you delay workup or treatment due to COVID testing?” and “Do you think other medical staff tried to avoid performing such procedures?” were considered to evaluate the impact of the COVID-19 pandemic on the workflow. A univariable logistic regression was performed to identify the variables associated with the outcome of interest. Sex, the estimated number of FBs ingested, the estimated number of FBs aspirated, and the CAS were considered explanatory variables of clinical relevance.

Statistical significance was established for a *p* value less than 0.05. Analyses were performed using the R System [31].

## 3. Results

A total of 58 physicians responded to the survey, including 18 (32%) from Europe, 16 (28%) from South America, 12 (21%) from Asia, and the rest from North America, Australia, and Africa. With this sample size, the precision of proportion estimates is at least 12.85%, calculated as the half-width of the 95% confidence interval and assuming maximum variability (i.e., an expected probability of 0.5). The respondents were more often men (70%) with a median of 16 years of experience.

### 3.1. COVID-19-Related Questions Results

Table 1 shows the answers to the COVID-19-related questions according to the years of experience category (≤16 years, >16 years).

Respondents reported that the estimated number of aspirated FBs was lower or the same (43, 74%) as the number of ingested FBs (43, 74%). There were no significant differences between the two groups regarding the COVID-19-related questions. A total of 43 of the 58 (74%) respondents did not report delays in procedures due to COVID-19; however, 14 respondents (24%) reported concerns. The number of cases with diagnostic delays was 15. In nine cases (60%), the delay was estimated to be one day, and in six cases (40%), it was estimated to be one hour. Fifty-two respondents (90%) reported that children with suspected FBs did not undergo different procedures. Thirty (52%) physicians reported having to delay the procedure or treatment due to a child’s COVID-19 test results. Twenty-five (43%) of the respondents declared that some of the medical staff tried to avoid performing medical procedures, and two (3%) successfully avoided them. Most of the respondents (54; 93%) reported that personal protective equipment (PPE) was available in their structure; notably, 31 (55%) of them thought that the use of PPE complicated the procedure.

Thirteen physicians (22%) routinely checked their COVID-19 status after each procedure, and the health agency recommended 23 (40%) of these checks. Eight respondents (11%) with COVID-19 swabs before the procedure reported a change in the workup.

### 3.2. The CAS questionnaire Results

The CAS was low and slightly, but not significantly, varying according to COVID-19 positivity (Table 2).

### 3.3. Results of Univariable Models

No predictors of delays in routine FB management and avoidance of medical procedures were identified in univariable logistic regression (Table 3).

## 4. Discussion

The purpose of this study was to evaluate the effects of COVID-19 on the activity workflow of physicians involved in the management of pediatric FB injury patients and to evaluate their level of anxiety during the pandemic. The investigation was based on the perceptions of field experts.

Regarding the volume of activities in the ENT department, our survey showed that for 43 (74%) of the respondents, the number of aspirated or ingested FBs was the same or lower. This result aligns with a study conducted in Greece that reported a total reduction in the number of presentations in all cases, the most negligible reduction being in cases of foreign bodies [32]. In contrast, the study of Pizzol et al. [33] in an Italian pediatric referral center showed an increase in the number of battery ingestions during the COVID-19 lockdown. In an Indian tertiary care center, cases of nasal FBs were more complicated [34]. In the Canary Islands, a decrease of 66.75% (95% CI: −65.6; −67.7; *p* < 0.001) was reported for ED visits, with an increase in the number of cases of ingested foreign bodies and intoxication [35]. Ozturk [36] compared the clinical course and management of bronchoscopy for suspected foreign body aspiration in children. In their results, bronchoscopy for FB aspiration was performed in a higher proportion of FBA cases (59% vs. 38%), and the procedure was performed in a higher number of younger infants in the pandemic period compared to the pre-pandemic period. Additionally, in a study by Neal et al. [37], the rate of FB ingestions one year after the declaration of the COVID-19 pandemic was compared with that of the previous three years. The total number of pediatric ED admissions decreased, whereas the number of patients presenting with FB ingestion increased. In the context of ENT emergencies in Spain, there was a notable decline of nearly 50% in ENT emergency department visits during the initial wave of COVID-19 [38]. Although the nature of most cases in the COVID-19 era remained consistent with the pre-COVID-19 era, it is important to note that this analysis encompassed all visits to the ENT emergency department. The fact that the number of cases of FB injuries did not decrease could be explained by the long-term effects of home isolation due to COVID-19 restrictions and the fact that FB injuries often require emergency procedures that cannot be delayed. As in the experience of Palas et al. [39], interventions were recommended only in emergency procedures using appropriate PPE. Another aspect to be taken into consideration is the fact that in all the experiences reported, the total number of ED admissions decreased, and the proportion of FB injuries increased or remained stable, which could have been driven, as suggested in the study of Neal et al. [37], by the overall decrease in the volume of ED admissions. Moreover, our survey was distributed in the first wave of the pandemic, whereas other studies included broader periods, such as 11 March–25 June 2020, in a study by Wallis Gómez et al. [35].

The guidelines of the French Association for Pediatric Otorhinolaryngology (AFOP) and the French Society of Otorhinolaryngology (SFORL) suggest performing a CT scan to confirm indications for endoscopy [40]. In our survey, only six respondents (10%) reported a higher number of CT scans to assess FB injury.

Delayed presentation to the hospital was reported in other works [20,21]. For respondents in the present survey, delayed emergency department visits were a problem that was reported by 26% of the sample. Another issue evaluated by the survey was the delay in medical workups for suspected FBs, which was reported by 14 (24%) of the respondents. For 28 (48%) of the respondents, delays in examination or treatment of children were related to COVID test results.

After the beginning of the pandemic, there was a shortage of PPE. Several countries and organizations took various actions, such as requesting product exclusions from tariffs, managing PPE supplies, requisitioning domestic production, imposing export restrictions, and invoking the Defense Production Act to address the shortage and secure PPE for health care workers. Additionally, there were efforts from the US and other countries to procure or import large quantities of PPE to increase their strategic stockpiles [41]. However, the availability of PPE, which is needed primarily for flexible nasal endoscopy [40], was not found to be an issue in the present study. The survey respondents reported that PPE was available to all staff in 93% of the cases; however, the use of PPE complicated procedures for half of the survey respondents. For 35 (60%) of the respondents, procedures took longer after rather than before the pandemic due to the use of PPE.

In the literature, the prevalence of stress, anxiety, and depression, especially among front-line health care workers caring for COVID-19 patients, ranges from 23.2% in the review of Pappa et al. [42] to 25.8% (95% CI 20.5–31.9%) in the meta-analysis of Salari et al. [43]. In another cross-sectional study in a Danish primary ENT practice, the authors found that 13% of health care workers had signs of anxiety [44]. However, in this international survey, otorhinolaryngologists reported a low level of anxiety according to their CAS scores. Only a few survey respondents reached the threshold proposed by the authors [26]. To our knowledge, this is the only study that evaluated anxiety levels during the COVID-19 pandemic in this population within different countries.

A web-based survey conducted in April 2020 in Oman showed that young and female physicians experience a greater impact on their mental health [45]. The study by Li et al. evaluated mental health in young physicians from 12 hospitals with the Generalized Anxiety Disorder-7 scale for anxiety and the Patient Health Questionnaire-9 for depression and found that they experienced an increase in these symptoms after the COVID-19 outbreak [46]. Furthermore, survey respondents showed low anxiety levels during the pandemic; even when considering different levels of experience, no differences were revealed.

### Limitations

Although it adopts an international perspective, this study primarily relied on the subjective accounts of otorhinolaryngologists. It is important to acknowledge the potential for bias in participant self-selection, as those who chose to respond to the survey may have been more predisposed to do so due to their heightened awareness of the significance of sharing experiences, stemming from their scholarly engagement in the Susy Safe working group. Furthermore, it is worth noting that the participants in this study had diverse specialties and worked in a variety of clinical settings.

Moreover, another limitation of this study is the relatively small sample size, which comprised 58 subjects out of the 172 physicians within the Susy Safe network.

## 5. Conclusions

The present survey offers insights into the experiences of otorhinolaryngologists from various countries during the first wave of the pandemic. In specific settings, such as the management of foreign body injuries in children, workflows, especially in emergency departments, remained largely unchanged. These injuries often demand immediate attention without prior planning. The survey findings highlight a strong adherence to international guidelines, particularly regarding the use of personal protective equipment (PPE) and containment measures. Notably, COVID-19 did not significantly disrupt patient trajectories or treatment plans, and physicians did not appear to express heightened concerns about COVID-19 infection, despite the adoption of robust personal protection and some degree of patient protection measures. These results are encouraging, especially considering the potential risk of negative impacts on everyday clinical practices due to pandemic-related work overload, which has been extensively documented in other health care domains.

## Figures and Tables

**Table 1:** COVID-19-related questions according to years of experience (≤16 years, >16 years) of the otorhinolaryngologists. Data are reported as percentages (absolute numbers) for categorical variables.

Questions		N	≤16 Years	>16 Years	Overall	*p* Value
			(N = 30)	(N = 28)	(N = 58)	
Aspirated FB (N)	Higher	58	17% (5)	36% (10)	26% (15)	0.098
	Lower or the same		83% (25)	64% (18)	74% (43)	
Ingested FB (N)	Higher	58	20% (6)	32% (9)	26% (15)	0.291
	Lower or the same		80% (24)	68% (19)	74% (43)	
Different type	No	58	90% (27)	89% (25)	90% (52)	0.929
	Yes		10% (3)	11% (3)	10% (6)	
Delayed presentation to the hospital	No	58	70% (21)	79% (22)	74% (43)	0.456
	Yes		30% (9)	21% (6)	26% (15)	
Worried about the delay	No	15	11% (1)	0% (0)	7% (1)	0.398
	Yes		89% (8)	100% (6)	93% (14)	
Estimated delay	Days	15	78% (7)	33% (2)	60% (9)	0.085
	Hours		22% (2)	67% (4)	40% (6)	
Delayed medical examination for a suspected FB	No	58	73% (22)	79% (22)	76% (44)	0.641
	Yes		27% (8)	21% (6)	24% (14)	
Different Work for Children with suspected of FB injury	No	58	73% (22)	89% (25)	81% (47)	0.121
	Yes		27% (8)	11% (3)	19% (11)	
Use of more CT scans to evaluate FB injury	No	58	83% (25)	96% (27)	90% (52)	0.102
	Yes		17% (5)	4% (1)	10% (6)	
Delayed workup/treatment due to COVID-19 test results	No	58	57% (17)	46% (13)	52% (30)	0.436
	Yes		43% (13)	54% (15)	48% (28)	
Other medical personnel tried to avoid performing procedures	No	58	47% (14)	68% (19)	57% (33)	0.103
	Yes		53% (16)	32% (9)	43% (25)	
Did you personally try to avoid performing such procedures?	No	57	93% (28)	96% (26)	95% (54)	0.617
	Yes		7% (2)	4% (1)	5% (3)	
Referred COVID cases to another center	No	58	97% (29)	96% (27)	97% (56)	0.96
	Yes		3% (1)	4% (1)	3% (2)	
Availability of PPE for all staff for FB removal procedures	No	58	7% (2)	7% (2)	7% (4)	0.943
	Yes		93% (28)	93% (26)	93% (54)	
Use of PPE during FB removal procedures	No	58	10% (3)	4% (1)	7% (4)	0.334
	Yes		90% (27)	96% (27)	93% (54)	
The use of PPE complicated the procedures	No	56	34% (10)	56% (15)	45% (25)	0.113
	Yes		66% (19)	44% (12)	55% (31)	
The procedures took more time than before	No	58	33% (10)	46% (13)	40% (23)	0.308
	Yes		67% (20)	54% (15)	60% (35)	
Use of different protocols for anesthetizing distinct cases	No	58	80% (24)	79% (22)	79% (46)	0.893
	Yes		20% (6)	21% (6)	21% (12)	
Different anesthetizing procedures	Avoid spontaneous breathing	10	0% (0)	67% (4)	40% (4)	0.035
	Physical protection		100% (4)	33% (2)	60% (6)	
Different surgical techniques for clinical cases	No	58	93% (28)	93% (26)	93% (54)	0.943
	Yes		7% (2)	7% (2)	7% (4)	
Higher number of complications	No	58	97% (29)	96% (27)	97% (56)	0.96
	Yes		3% (1)	4% (1)	3% (2)	
Routinely checked COVID-19 status after procedures	No	58	80% (24)	75% (21)	78% (45)	0.648
	Yes		20% (6)	25% (7)	22% (13)	
The center checked the COVID-19 status	No	57	57% (17)	63% (17)	60% (34)	0.629
	Yes		43% (13)	37% (10)	40% (23)	
Awareness of performing procedures in COVID-19-positive patients	No	58	70% (21)	68% (19)	69% (40)	0.86
	Yes		30% (9)	32% (9)	31% (18)	
COVID-19 positivity	No	58	90% (27)	82% (23)	86% (50)	0.386
	Yes		10% (3)	18% (5)	14% (8)	
COVID-19 positivity due to FB removal procedures	No	8	67% (2)	80% (4)	75% (6)	0.673
	Yes		33% (1)	20% (1)	25% (2)	
COVID-19 positivity among staff due to FB removal procedures	No	58	100% (30)	100% (28)	100% (58)	
	Yes		0% (0)	0% (0)	0% (0)	
Isolation after procedures on COVID-19-positive patients	No	58	87% (26)	100% (28)	93% (54)	0.045
	Yes		13% (4)	0% (0)	7% (4)	
Preferences of other staff members regarding performing procedures on COVID-19-positive patients	No	58	87% (26)	86% (24)	86% (50)	0.916
	Yes		13% (4)	14% (4)	14% (8)	
Change of workup	Swab for COVID-19	11	100% (8)	100% (3)	100% (11)	

Abbreviations: FB = foreign body, CT = computed tomography, PPE = personal protective equipment.

**Table 2:** Descriptive results of the CAS according to COVID-19 positivity.

Questions	Levels	N	No (N = 50)	Yes (N = 8)	Combined (N = 58)	*p* Value
I felt dizzy, lightheaded, or faint when I read or listened to FB cases	For more than 7 days	56	2% (1)	0% (0)	2% (1)	0.021
	Not at all		94% (45)	62% (5)	89% (50)	
	Rare, less than one or two days		2% (1)	25% (2)	5% (3)	
	Several days		2% (1)	12% (1)	4% (2)	
I had trouble falling or staying asleep because I was thinking about FB cases	More than 7 days	57	2% (1)	0% (0)	2% (1)	0.888
	Not at all		85% (41)	88% (7)	86% (48)	
	Rare, less than one or two days		8% (4)	12% (1)	9% (5)	
	Several days		4% (2)	0% (0)	4% (2)	
I felt paralyzed or frozen when thinking about or being exposed to information about FB cases	Not at all	57	96% (46)	62% (5)	91% (51)	0.002
	Rare, less than one or two days		4% (2)	38% (3)	9% (5)	
I lost interest in eating when I thought about or was exposed to information about FB cases	Not at all	56	96% (46)	88% (7)	95% (53)	0.332
	Rare, less than one or two days		4% (2)	12% (1)	5% (3)	
I felt nauseous or had stomach problems when I thought about or was exposed to information on FB cases	More than 7 days	56	2% (1)	0% (0)	2% (1)	0.1
	Not at all		94% (45)	75% (6)	91% (51)	
	Rare, less than one or two days		4% (2)	25% (2)	7% (4)	

**Table 3:** Results of the univariable models. For each variable, odds ratios are reported with 95% confidence intervals (95% CIs) and *p* values.

		Did you Delay Workup or Treatment due to COVID Test Results?			Do you Think other Medical Personnel Tried to Avoid Performing Such Procedures?			
Predictors	N	Odds Ratio	95% CI	*p* Value	N	Odds Ratio	95% CI	*p* Value
Indicate your sex (male)	57	4.4	1.30–17.84	0.024	57	0.75	0.24–2.39	0.622
The estimated number of aspirated FBs was [Lower or the same]	58	1.57	0.48–5.42	0.458	58	0.82	0.25–2.74	0.746
The estimated number of ingested FBs was [Less than or equal to]	56	1.09	0.33–3.63	0.885	58	0.57	0.17–1.87	0.355
COVID-19 Anxiety Score	56	1.33	0.90–2.22	0.197	56	1.42	0.95–2.37	0.116

## Data Availability

The data presented in this study are available upon request from the corresponding author. The data are not publicly available due to privacy.
